# ROS Scavenging and Osteogenic Differentiation Potential of L-Methionine-Substituted Poly(Organophosphazene) Electrospun Fibers

**DOI:** 10.3390/biomimetics9110676

**Published:** 2024-11-06

**Authors:** Meng Wang, Kibret Mequanint

**Affiliations:** Department of Chemical and Biochemical Engineering, The University of Western Ontario, London, ON N6A 5B9, Canada; mwang529@uwo.ca

**Keywords:** biodegradable poly(organophosphazenes), ROS scavenger, material-guided cell behavior, human mesenchymal stem cells, vascular smooth muscle cells, vascular tissue engineering, electrospinning

## Abstract

This study investigated the application of poly[bis (ethylmethionato) phosphazene] (PαAPz-M) electrospun fibers in tissue engineering, focusing on their reactive oxygen species (ROS) scavenging capabilities and material-directed cell behavior, including the influence of their degradation products on cell viability and differentiation, and the scaffold topography’s influence on cell alignment. The ROS scavenging ability of PαAPz-M was assessed by DPPH assay, and then PαAPz-M’s protection against exogenous ROS was studied. The results showed enhanced cell viability on PαAPz-M fiber mats under oxidative stress conditions. This study also investigated the effects of the degradation products of PαAPz-M on cell viability and osteogenic differentiation. It was observed that the late-stage degradation product, phosphoric acid, can significantly influence the osteogenic differentiation of MSCs. In contrast, methionine, which is the early-stage degradation product, showed a minimal influence. Additionally, the study fabricated fiber mats that can lead to enhanced cell alignment while maintaining high porosity. Collectively, this study expanded the applications of PαAPz-M fiber mat protection against oxidative stress and guiding osteogenic differentiation and cell alignment.

## 1. Introduction

Although biodegradable synthetic polymers such as polylactic acid, polycaprolactone, poly(lactic-co-glycolic acid) and their composites have been studied as temporary scaffolds in tissue engineering, they generally lack the biomimetic properties. As tissue engineering evolves, the demands placed on biodegradable materials extend beyond their biocompatibility and biodegradation properties and should also include the biomimetic aspects of natural materials. Researchers are now seeking materials that can meet unique biomimetic requirements, such as signaling capability, mechanical properties, and dynamic adaptability [1]. Biomimetic electrospun fibers replicate key features of the natural extracellular matrix (ECM), such as a large surface area, good porosity, and a good nanoscale architecture, promoting cell adhesion, proliferation, and differentiation and thus providing a supportive environment for tissue regeneration. Electrospun amino acid-based polyphosphazene fibers are innovative biomimetic materials that combine the advantages of amino acids and polyphosphazenes, offering unique properties for tissue engineering and biomedical applications. This versatile class of polymers with a flexible backbone made of alternating phosphorus and nitrogen atoms, which can be functionalized with a variety of side groups, including amino acids, offers enhanced biocompatibility, biodegradability, and bioactivity, making these fibers ideal for mimicking the ECM [2]. The incorporation of amino acids, such as methionine, into the polyphosphazene structure further improves the bioactivity of the fibers by facilitating interactions with cells and proteins. Polyphosphazenes containing α-amino acids such as PαAPz-M have shown good viability of cells and differentiation with the supplementation of different biochemical induction factors [3]. Most importantly, the amino acid L-methionine is a direct target of reactive oxygen species (ROS) to protect cells from oxidative stress [4]. However, the possible contribution of its thioether structure in tissue engineering and the biochemical and biophysical cues provided by the scaffold have not been previously studied.

One of the most important functions of methionine in the body is to counteract oxidative stress [5]. Reactive oxygen species (ROS), including peroxides and free radicals, are crucial molecules in physiological processes. Nevertheless, they can detrimentally modify cellular components, impairing DNA and proteins and, consequently, obstructing new tissue formation when overexpressed [6,7]. However, even when using biocompatible materials, implantation surgery can lead to trauma, foreign body reactions, and localized inflammation, resulting in the significant production of ROS [8].

While small molecules have been met with limited success in vivo due to their transient presence, research on ROS scavengers focuses more on macromolecular polymers [6,9]. Among them, poly(organophosphazenes), due to their biodegradability and easily tailored properties, are believed to be involved in ROS scavenging. As an example, the inclusion of aniline tetramer onto the glycine-based phosphazene produced a biodegradable polymer with ROS scavenging capability [10]. Considering that the pure amino acid methionine is known to scavenge ROS [11,12], and PαAPz-M showed good biocompatibility [3], they may serve to modulate the ROS level without impairing cellular physiological processes.

In addition to traditional approaches, which mainly rely on the in vitro culture supplementation of different biochemical induction factors, material-directed differentiation, such as substrate stiffness, surface topography, and material composition, have recently been used as alternative regulators for MSC differentiation [13]. An increasing expression of vascular smooth muscle cell markers in MSCs on the PαAPz-M fiber mat was observed in the presence of growth factors [3]. However, phosphoric acid, a primary degradation product of PαAPz-M, has demonstrated potential in guiding the osteogenic differentiation of MSCs [14,15]. Additionally, the influence of ROS on cell differentiation has been widely discussed [16,17].

Meanwhile, cell alignment is present in numerous tissues, including heart valves, blood vessels, tendons, and nerves [18,19,20,21]. Electrospun and aligned fibers are considered to be ideal candidates for producing topographical features that guide cell arrangements and growth [22,23]. Although several strategies have been developed to produce aligned electrospun fibers, each method has limitations and material requirements, and the alignment depends highly on the system parameters, such as voltage and solution viscosity [24,25].

This study aimed to explore the application of PαAPz-M for tissue engineering in terms of ROS scavenging ability and cell behavior under the influence of an exogenous ROS-generating compound. Furthermore, material-directed differentiation based on PαAPz-M, focusing on how PαAPz-M’s degradation products and the topography of fiber mats can guide MSC differentiation without growth factors, were studied.

## 2. Materials and Methods

### 2.1. Materials

Poly(ester amide) (PEA) and PαAPz-M were synthesized following previous reports. PEA, specifically 8-Phe-4, was synthesized by interfacial polymerization using sebacoyl chloride, butanediol, and L-phenylalanine as the amino acids, according to previous publications [3,26]. PαAPz-M was synthesized by thermal ring-opening polymerization with hexachlorocyclotriphosphazene and macromolecular substitution with L-methionine ethyl ester hydrochloride (H-Met-OEt·HCl).

Anhydrous tetrahydrofuran (THF) was purchased from Caledon Labs (Georgetown, ON, USA) and chloroform (CHCl_3_) was purchased from Sigma Aldrich (Milwaukee, WI, USA). The 30% hydrogen peroxide (H_2_O_2_) used as the exogenous ROS was purchased from Fisher Chemical (Fair Lawn, NJ, USA). L-methionine ethyl ester (L-Met-Et) was obtained from Alfa Aesar (Ward Hill, MA, USA). Sodium phosphate was obtained from Sigma (Milwaukee, WI, USA).

### 2.2. Preparing Electrospun Fibrous Scaffolds

Table 1 presents the parameters, including voltage, concentration, flow rate, and rotating speed, for the preparation of electrospun fibrous scaffolds. The other electrospinning parameters, including a 20 cm distance to the collector and a nozzle size of 22 G, were maintained.

### 2.3. Characterizations with SEM

The morphologies of all fiber mats were characterized using a scanning electron microscope (SEM) (Model S-3400N, Hitachi, Ltd., Tokyo, Japan). The samples were affixed to a stainless steel holder using carbon adhesive tabs and subsequently coated with a 5 nm layer of osmium using a Filgen OPC80T Osmium Plasma Coater (Filgen Nanosciences & Biosciences Inc., Jonoyama, Japan). SEM images were captured at an acceleration voltage of 5 kV, utilizing the microscope’s internal lens.

### 2.4. Cell Culture 

This study used mesenchymal stem cells derived from induced pluripotent stem cells (hereinafter named iMSC) generously provided by Dr. Dale Laird of Western University, London, ON, Canada. The fiber mat scaffolds, supported by aluminum foil, were carefully cut and their edges folded to secure the mats, creating an effective area of approximately 1 cm^2^. These prepared fiber mats were individually placed in the wells of a 24-well plate and sterilized using 70% ethanol for a minimum of 30 min, followed by triple rinsing with HBSS to prepare for cell seeding.

Before seeding the iMSCs, the sterilized fiber mats were coated with a 0.1% gelatin solution and incubated at 37 °C for 1 h. The iMSCs were then seeded onto these gelatin-coated fiber mats at 20,000 cells/cm^2^ density. The cells were cultured in mesenchymal stem cell expansion media (MSCEM, Cedarlane Labs, Burlington, ON, Canada; HMSC.E. MEDIA-450), supplemented with 10% fetal bovine serum, 1% L-glutamine, and 1% penicillin/streptomycin (all sourced from Fisher Scientific, Whitby, ON, Canada). The culture was maintained at 37 °C in a humidified incubator with 5% CO_2_.

### 2.5. ROS Scavenging Assays

A 2,2-Diphenyl-1-picrylhydrazyl (DPPH) scavenging assay was used to evaluate the ROS scavenging ability. DPPH (Sigma Aldrich, Milwaukee, WI, USA) was dissolved in ethanol at 5 mg/100 mL, then 0.1 g of the solid PαAPz-M or PEA or 1 cm×1 cm PαAPz-M or PEA fiber mats was immersed into 1.5 mL of the DPPH solution and kept in a dark for 2 h. The DPPH solution added to a 1 × 1 cm aluminum foil was used as the control. Then, the absorbance of the solution was measured by a UV–vis spectrophotometer (F-7000, Hitachi, Japan) between 400 and 800 nm. DPPH scavenging was calculated as follows:

(1) $$\mathrm{DPPH}\;\mathrm{scavenging}=\frac{A_{c}-A_{s}}{A_{c}}$$

where A_c_ is the absorbance of the control DPPH solution and A_s_ is the absorbance of the DPPH solution with biomaterials.

To evaluate the cell protection effect of the materials from exogenously generated ROS, iMSCs were cultured on aluminum foil, a PEA fiber mat, and a PαAPz-M fiber mat. Once the cells achieved approximately 95% confluence on the material surfaces around 5 days after seeding, H_2_O_2_ at concentrations of 1, 2, 5, and 10 mM was added into the culture medium. After a 2 h exposure to the H_2_O_2_-conditioned medium, the LIVE/DEAD™ Cell Imaging Kit (488/570) (Life Technologies, Burlington, ON, Canada) was used to stain the cells on the scaffolds. Then, the fluorescent images were captured and analyzed quantitatively using ImageJ software (version 1.50i). The live/dead stained cells were further counted to calculate cell viability.

### 2.6. Osteogenic Differentiation with Degradation Products

In performing the osteoinductive culture, cell cultures with and without scaffold were applied. iMSCs were seeded on the 24-well plates at a density of 20,000 cells/cm^2^ in all cases. When the cells reached 80% confluency, stock solutions with different degradation components were added to the medium. All stock solutions were adjusted to a pH of 7.4. The osteoinductive culture continued for 7 days.

### 2.7. RNA Extraction and Quantitative Real-Time qPCR

In total, 1 mL of TRIzol™ Reagent (Life Technologies Inc., Burlington, ON, Canada) was utilized to extract the total RNA from each sample for the qPCR assay. The cells were lysed for 10 min at room temperature, and then chloroform was added at a ratio of 1:5 (chloroform: Trizol). The samples were vortexed for 15 s and incubated at room temperature for 15 min. They were then centrifuged at 4 °C and 12,000× *g* for 15 min. The organic phase was removed, and the aqueous phase was transferred to another Eppendorf tube. Isopropanol was added at a ratio of 1:2 (isopropanol: Trizol) and incubated at room temperature for 10 min, followed by centrifugation at 12,000× *g* for another 10 min at 4 °C. The isopropanol was then aspirated, and the pellet was resuspended in 75% EtOH at a ratio of 1:2 (EtOH: Trizol) and centrifuged at 7500× *g* for 5 min at 4 °C. This final step was repeated twice to wash off the excess salts. After removing the EtOH, the pellet was air-dried, dissolved in 25 µL of DEPC water, and quantified with nanodrop (Thermo Scientific, Waltham, MA, USA).

The Promega™ Random Hexamers protocol (Fisher Scientific, Whitby, ON, Canada) was used to reverse transcribe 1 μg of total RNA into complementary DNA (cDNA). qRT-PCR was performed four times using a CFX96™ Real-Time System (C1000 Touch Thermal Cycler; Bio-Rad, Mississauga, ON, Canada), and human genes of interest were determined with SsoAdvanced Universal SYBR^®^ Green Supermix (Bio-Rad) as per the recommended procedures.

The primer sequences were as follows: Human Alpl forward primer 5′-CCT TCA CGG CCA TCC TAT ATG-3′, reverse primer 5′-CTG GTA GTT GTT GTG AGC GTA-3′. Human Runx2 forward primer 5′-CAC TGG GTC ACA CGT ATG ATT-3′, reverse primer 5′-AGG GAA GGG TTG GTT AGT ACA-3′. Human Sp7 forward primer 5′-GCC AGT AAT CTT CAA GCC AGA-3′, reverse primer 5′-CCA TAG TGA GCT TCT TCC TGG C-3′. Human OCN forward primer 5′-CTG CAT TCT GCC TCT CTG AC-3′, reverse primer 5′-CTA TTC ACC ACC TTA CTG CCC-3′. Human 18S forward primer 5′-GCG GTT CTA TTT TGT TGG TTT-3′, reverse primer 5′-CTC CGA CTT TCG TTC TTG ATT-3′. The results were analyzed using the comparative threshold cycle method and normalized with human 18S as an endogenous reference.

### 2.8. Immunofluorescence Microscopy

The cells were treated with 10% formalin for 10 min at room temperature and then washed three times with PBS. To enable intracellular staining, 0.1% Triton X-100 permeated all the samples for 10 min. Subsequently, for 30 min at room temperature, the cells were blocked in 2% BSA containing 22.52 mg/mL of glycine in PBS. For nuclear labeling, 4′6-diamidino-2-phenylindole (DAPI), obtained from Life Technologies, Canada, was used at a concentration of 300 nM in PBS. The coverslips were mounted onto microscope slides using PermaFluor™ Mounting Medium by Fisher Scientific™, Whitby, ON, Canada, and sealed with clear nail enamel. Images were captured using a 20x objective lens with Leica DMi8 (Leica Microsystems, Wetzlar, Germany) and analyzed using Leica Application Suite X (LAS X, version 3.7.5.24914) software.

### 2.9. Statistical Analysis

Where applicable, data are presented as mean ± standard deviation (SD) for *n* = 3. Tukey’s test was used for statistical analysis with one-way variance analysis. Statistical analysis of the data was performed using Origin. Differences were tested by one-way ANOVA, and a *p*-value of <0.05 was used for statistical significance.

## 3. Results and Discussion

### 3.1. Evaluation of ROS Scavenging Capacity of PαAPz-M

**Figure 1:**
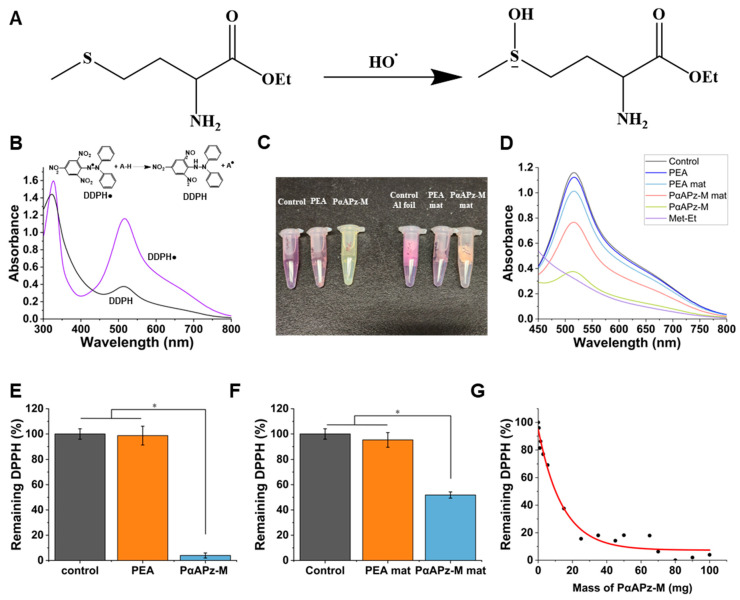
(**A**) The mechanism of L-Methionine derivative (L-Methionine ethyl ester) ROS scavenging. (**B**) The reaction of 2,2-diphenyl-1-picrylhydrazyl (DPPH) with an antioxidant. A-H = antioxidant radical scavenger; A● = antioxidant radical. (**C**) The DPPH solution (5 mg/100 mL, 1.5 mL) incubated with 100 mg of L-methionine, PEA, and PαAPz-M pellets, and 1 cm^2^ fiber mats for 2 h in the dark. (**D**) The absorbance of the corresponding solution after 2 h of incubation. The DPPH scavenging capacity of PEA and PαAPz-M pellets (**E**) and the corresponding fiber mats (**F**) based on absorption peaks at 510 nm. (**G**) The effect of PαAPz-M mass on DPPH scavenging. * *p* < 0.05.

ROS scavenging is the process by which cells neutralize harmful reactive molecules that can cause oxidative damage. The essential amino acid, L-methionine derivative (L-methionine ethyl ester), plays a role in antioxidant defense mechanisms by capturing free radicals. Figure 1A illustrates how methionine, the functional amino acid in PαAPz-M, scavenges ROS. The thioether group in methionine can capture ROS and be oxidized into the methionine sulphoxide [5]. Meanwhile, methionine sulphoxide can also be reverted, enabling continuous ROS regulation. The PαAPz-M synthesis only involves the amino groups of methionine. Therefore, the thioether structure is well-protected (Figure 1A). Modification of polyphosphazenes ester biomaterials with methionine may be a promising candidate for effective ROS scavenging. To evaluate this, the DPPH assay, which is a reliable method for assessing antioxidant activity, was employed. The DPPH assay measures the antioxidant activity of various substances based on the scavenging ability of antioxidants against the DPPH radical, which is a stable free radical. The DPPH radical (DPPH●), has a violet color (absorbance ~517 nm) due to the presence of an unpaired electron, which can be reduced when an antioxidant donates an electron (hydrogen) to the DPPH radical. This reduction (the formation of DPPH-H) leads to a color change from violet to a pale yellow. The reduction in color intensity (measured as a decrease in absorbance at 517 nm) indicates the scavenging activity of the antioxidant substance (Figure 1B). The greater the decrease in absorbance, the higher the antioxidant capacity of the tested substance, and the absorbance follows the Lambert–Beer law up to a 5 mM concentration [27].

The ROS scavenging capacities of pure L-methionine ethyl ester, pellets of PEA, and PαAPz-M, and the corresponding PEA and PαAPz-M fiber mats, are shown in Figure 1C. After incubation in the DPPH solution for 2 h, the characteristic color change caused by reactions of DPPH and the different materials was observed. For the control (DPPH only) and the PEA pellets, there was no color change, demonstrating that the PEA was not capturing the DPPH radical. However, for both PαAPz-M pellets and fibers, the pale yellow color was observed. This color change was further substantiated quantitatively using an absorption spectrophotometer at 517 nm, as shown in Figure 1D. Notably, both PEA and PEA fiber mats exhibited negligible deviation from the control, which means PEA did not have the capacity to scavenge ROS, while L-methionine, PαAPz-M pellets, and PαAPz-M fiber mats effectively neutralized DPPH. In the subsequent quantitative analysis of DPPH scavenging tests, both PαAPz-M pellets and PαAPz-M fiber mats demonstrated significant differences compared to the control and PEA, with nearly 100% and 50% DPPH scavenging abilities, respectively (Figure 1E,F). The difference between the pellet and fibrous forms of PαAPz-M was due to the mass used. In Figure 1E, the mass of PαAPz-M pellets used was 100 mg, but in Figure 1F, the fiber mass was ~10 mg (1 cm × 1 cm fiber mat). To validate this effect, we examined the relationship between varying masses of PαAPz-M and their DPPH scavenging capacity (Figure 1G). As can be seen, we found that a 1 cm × 1 cm fiber mat exhibited a similar DPPH scavenging ability equivalent to 10 mg of PαAPz-M pellets. This observed mass is notably higher than our estimated amount of PαAPz-M on the fiber mat (~5 mg), which can be attributed to the significantly larger surface area of the fiber mat, allowing for a more rapid and efficient reaction.

**Figure 2:**
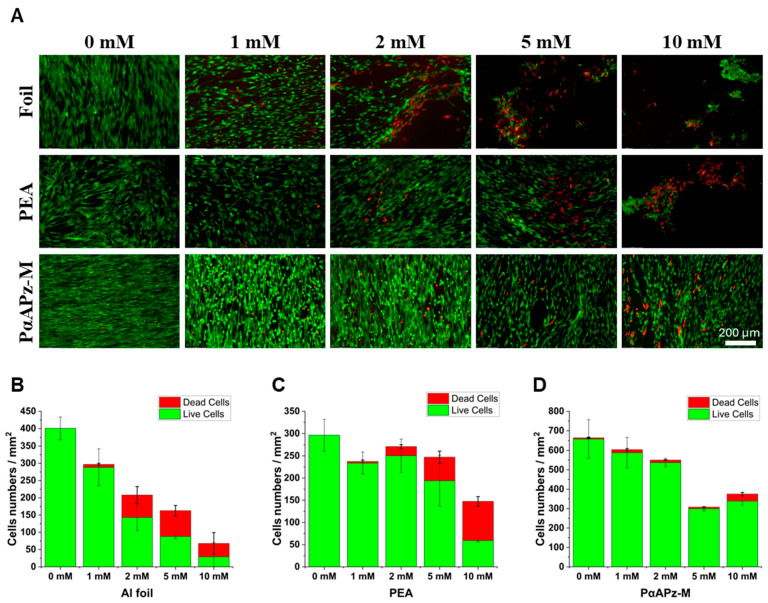
Evaluation of iMSC viabilities of fiber mats exposed to exogenous ROS. (**A**) Live/dead staining of iMSC cultured on each material for 5 days and treated with different concentrations of H_2_O_2_ for 2 h. Live cells are labeled in green and dead cells in red. The 200 µm scale bar shown on the PαAPz-M image at 10 mM is applicable to all images. Live and dead cell numbers per mm^2^ on Al foil (**B**), PEA mat (**C**), and PαAPz-M mat (**D**) based on live/dead staining.

After verifying the ROS scavenging capability of the PαAPz-M, its potential to protect cells from exogenous ROS was evaluated. Different concentrations of H_2_O_2_ were added to the culture media to simulate the exposure of exogenous ROS. Live/dead assays were used to qualify and quantify the viability of iMSC after the exposure. As shown in Figure 2A, with the increase in H_2_O_2_ concentration, increasing red fluorescence signals representing dead cells were observed, and the cell density decreased. Aluminum foil was used as a control since electrospun fibers were collected on the foil. In this control group, a notable reduction in cell density was observed upon exposure to 1 mM H_2_O_2_. At 2 mM, the detachment of cells from the foil was noted, and at concentrations over 5 mM, the control group exhibited almost complete cell detachment. Cell detachment on the PEA surface was less severe. Considerable detachment only occurred at concentrations over 10 mM. Although the DPPH assay indicating PEA did not scavenge ROS, the enhanced cell adhesion on PEA fiber mats can be attributed to the large surface area presented by the nanofiber mat structure. The viability of iMSCs on the PαAPz-M fiber mat showed a different scenario. At concentrations of 1 mM and 2 mM, some iMSCs adopted a rounded shape without reducing cell density. The cell density on PαAPz-M at 5 mM H_2_O_2_ resembled that of PEA and the control at 1 mM, thus demonstrating the ROS protection of the PαAPz-M material. For the PαAPz-M, cell death was noticeable only at 10 mM. With the exogenous ROS treatment, PαAPz-M fiber mats showed iMSC protection against oxidative damage. As shown in Figure 1B–D, fewer dead cells were encountered only on PαAPz-M fiber mats.

### 3.2. The Effect of Model Degradation Products on Cell Viability

The degradation of PαAPZ is through the hydrolysis of amino acid ester, the cleavage of side groups, and, ultimately, the breakdown of the backbone [28]. Therefore, the soluble degradation products of PαAPz-M should be composed of the corresponding side groups and ions, such as phosphate and ammonium. Although methionine, phosphate, and ammonium are commonly found in the human body, previous studies have indicated that these degradation products may accumulate in porous structures and produce a microenvironment that may influence cell behavior [29,30,31]. Therefore, it is essential to study the cell viability at high concentrations of these degradation products.

**Figure 3:**
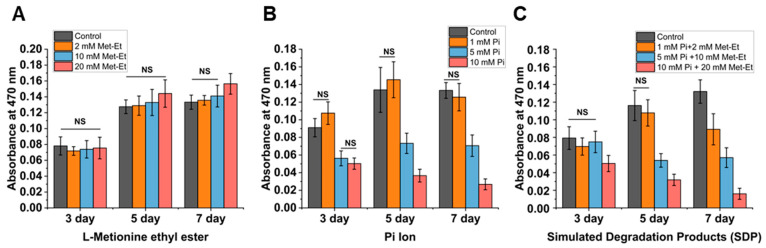
Cell viability of iMSCs cultured with media containing various concentrations of (**A**) Methionine, (**B**) Pi ion, and (**C**) simulated degradation product (SD) for 3–7 days, assessed using the MTT assay. After seeding the cells onto the culture plates, the additives were introduced to the media at the indicated concentrations. The stock solutions of each additive were prepared at concentrations of 1 M, with the pH adjusted to 7.4 post-preparation. The stock solutions were then diluted into the culture medium to achieve the desired target concentrations. Statistical significance was observed between groups, except for those labeled with “NS” (not significant).

To simulate the effects of the degradation products of PαAPz-M on iMSC behavior, aqueous solutions of sodium phosphate (source of Pi ions), L-methionine ethyl ester, and their 1:2 molar ratio simulated degradation product (SDP) on cell viability were compared. The 1:2 simulated molar ratio is based on the expected degradation of PαAPz-M (see the polymer repeat unit in Figure 5A). All stock solutions were adjusted to a pH of 7.4, and then, the stock solutions were added to the culture medium at 1, 5, and 10 mM concentrations. As shown in Figure 3, the metabolic activity with different treatments was tested using the MTT assay for 3, 5, and 7 days of iMSC culture. No significant change in cell metabolic activity was observed in the L-methionine ethyl ester group for the indicated times; however, 20 mM of L-methionine ethyl ester led to a slight increase in metabolic activity. In contrast, the groups supplemented with phosphate-only and phosphate-amino acid mixture show a significant decrease in metabolic activity when concentrations of phosphoric ions are at 5 mM and above.

**Figure 4:**
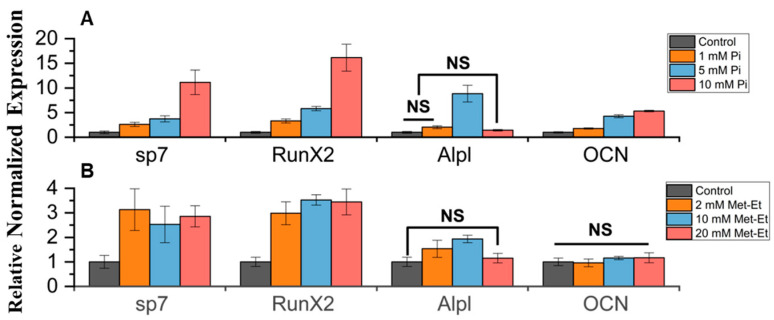
iMSC mRNA expression of SP7, RunX2, Alpl, and OCN was analyzed using RT-qPCR after cell treatment with (**A**) Pi ions and (**B**) Methionine for 7 days under 2D conventional tissue culture conditions. Differentiation was induced once the cells reached 80% confluence to ensure an adequate number of cells for differentiation. Within the indicated groups, statistical significance exists except for those labeled with NS (not significant).

Recent research indicates that high levels of phosphoric acid can substantially increase the expression of osteogenic markers [32,33,34]. Meanwhile, the ROS levels that methionine influences can also control osteogenic differentiation and calcium deposition [35,36]. Therefore, osteogenic differentiation studies of iMSC cultured in the presence of the expected degradation products of PαAPz-M will be insightful. To investigate the osteogenic differentiation of iMSC, the expression of osteogenic marker genes was analyzed under 2D conventional tissue culture conditions, and the results are shown in Figure 4. With the presence of high phosphate levels, there is a notable increase in the osteogenic markers RunX2, ALPL, SP7, and OCN (Figure 4A). The highest expression of Alpl is found at the concentration of 5 mM of Pi ions, which is similar to reports on phosphate’s effects on bone marrow mesenchymal stem cells [33,37]. This could be because Alpl plays a crucial role in early osteogenesis, hydrolyzing phosphates to promote cell maturation, and its expression diminishes following the completion of maturation [38]. However, the contribution of the L-methionine derivative to osteogenesis seems minimal (Figure 4B), and only a relatively slight increase in Sp7 and RunX2 was observed. Although SP7, RunX2, and OCN genes were upregulated in the 10 mM Pi ion group, poor cell viability diminishes the value of the gene expression data. However, compared to the control, the same genes were also upregulated at lower Pi concentrations where cell viability was high.

### 3.3. The Effect of the Degradation Products of PαAPz-M on Osteogenic Differentiation

**Figure 5:**
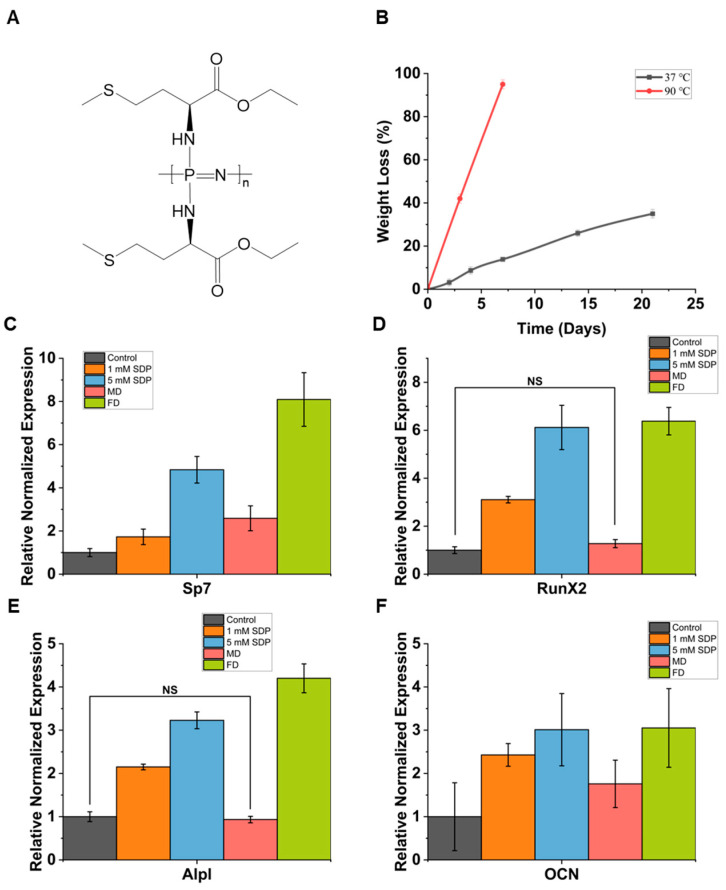
(**A**) The molecular structure of PαAPz-M. (**B**) The degradation behaviors of the PαAPz-M solid. Analysis of iMSC mRNA expression levels for (**C**) SP7, (**D**) RunX2, (**E**) Alpl, and (**F**) OCN using RT-qPCR. The cells were treated for 7 days on fiber mats with simulated degradation product (SDP), mid-staged degradation products (MDs), and fully degraded products (FDs). The concentrations of MDs and FDs were calculated based on the mass loss during degradation and the molecular weight of the PαAPz-M repeating unit. Prior to use, all stock solutions were pH-adjusted to 7.4 and prepared at concentrations significantly higher than the target concentration. Within the indicated groups, statistical significance exists except for those labeled with NS (not significant).

After studying the effects of Pi ions and L-methionine ethyl ester on cell viability and iMSC differentiation, the osteogenic differentiation of iMSC cultured on PαAPz-M fiber mats and treated with the staged degradation products of PαAPz-M was evaluated. Normally, the PαAPz-M fibers would degrade over time, releasing Pi ions and L-methionine derivatives to influence cell differentiation, but the L-methionine derivative in the PαAPz-M can protect cells from oxidative damage before polymer degradation (Figure 1 and Figure 2). In order to gain insight into the effect of the degradation products during short-term culture iMSC differentiation, we supplemented cultures with accelerated degradation products. As shown in Figure 5B, the PαAPz-M lost around 40% of its mass after 21 days of degradation at 37 °C, suggesting a slower degradation relative to the short cell culture time (7 days). However, PαAPz-M completely degraded at 90 °C in 7 days. We separately collected the mid-stage degradation (MD) soluble products, which occurred on day 4, and the full degradation (FD) soluble fraction, which happened on day 7. The degradation behavior based on the mass loss of PαAPz-M is very similar to previously reported trends, albeit with a slightly accelerated rate [39,40]. This difference could be attributed to the phosphazene polymerization method employed. The degradation products were added to the culture medium at 10 mM, based on the estimated phosphate-ion concentration derived from the mass loss of the material. iMSC, cultured on the PαAPz-M fiber mats, was used as a control. Furthermore, iMSC cultured on PαAPz-M fibers and treated with simulated degradation products (SDP) was used as an additional comparator.

Figure 5C–F show the expression of osteogenic markers SP7, RunX2, Alpl, and OCN by iMSCs after a 7-day culture period with supplemented degradation products on the fiber mats. Compared to the control (iMSCs cultured on PαAPz-M fibers), except OCN, all of the tested genes were significantly upregulated when the simulated degradation products (SDPs) were used. The concentrations of the SDPs used as comparators were based on the expected concentrations of the phosphate and L-methionine derivatives that will be released. Thus, 1 mM SDP has a ratio of 1:2 (Pi:Met-Et). With regard to the actual degradation products, only SP7 was significantly upregulated in response to the mid-stage degradation (MD) composition compared to the control. However, three of the four tested genes were significantly upregulated in response to final-stage degradation (FD) product supplementation.

The different effects of the mid- and late-stage degradation products on iMSC differentiation can contribute to the staged degradation behavior of polyphosphazene, wherein PαAPz initially releases amino acid groups, followed by the subsequent breakdown of its backbone, which will release inorganic phosphates [40,41]. Therefore, in the early stages of degradation, PαAPz-M and its degradation products, which are mainly composed of L-methionine, will not guide stem cells to osteogenic differentiation. In the past, polyphosphazene was widely studied in bone tissue engineering due to its phosphate-containing degradation products. This may also explain why polyphosphazenes have mainly been used in bone tissue engineering.

Meanwhile, soft tissue calcification is often associated with pathological conditions, and it is important to recognize that such calcification can also be a natural healing response to injury [42]. Indeed, in certain scenarios within the circulatory system, such phosphate-guided calcification may play a beneficial role as well [32,43]. Therefore, the ability of PαAPz-M to guide osteogenic differentiation, as demonstrated in these experiments, is an important advantage of its application in bone tissue engineering, but it will not hinder its application in other tissues.

### 3.4. Cell Orientation on Aligned Electrospun Fibers

After studying PαAPz-M-directed ROS scavenging effects and iMSC differentiation, the topological cue that could potentially be provided by the fiber mat was further tested. In this study, aligned electrospun fibers were prepared with higher flow rates and lower voltages [44]. This is because the increased fiber diameter and reduced electric field intensity can mitigate the impact of the bending zone on fiber arrangement. To further reduce the impact of the bending zone, the PαAPz-M and PEA were blended to increase the strength of the fiber. After optimizing the electrospinning parameters, the aligned fibers were produced following the parameters in Table 1.

**Figure 6:**
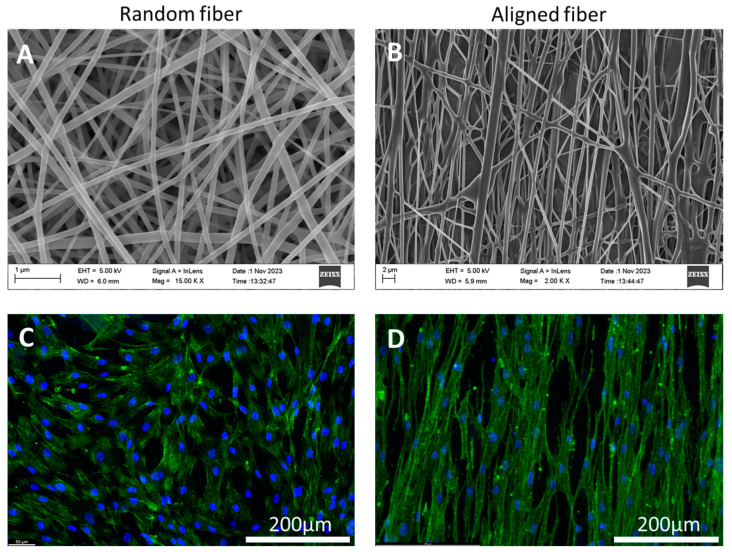
SEM images of electrospun (**A**) random and (**B**) aligned fibers from the blend of PEA and PαAPz-M. Fluorescence images of iMSC on (**C**) random and (**D**) aligned fibers. After 7 days of culturing, F-actin is labeled by Phalloidin in green and nuclei are labeled by DAPI in blue (**C**,**D**).

The SEM image and the corresponding cell morphology are shown in Figure 6. As shown in Figure 6B, a rise in the flow rate corresponded with an increase in fiber diameter (compare scale bars in A and B), producing fiber mats composed of aligned micron-scale fibers interspersed with randomly distributed nanofibers. After producing the aligned fiber mats, cells on both random and aligned fiber mats were stained with immunofluorescence to study the effect of scaffold topography on cell–scaffold interactions. As shown in Figure 6C,D, it is evident that cells on aligned fiber mats displayed an aligned arrangement, while on the random fiber mats, cells exhibited a random distribution. This indicates that, within the structure of electrospun fiber mats, part of the aligned micro-sized fibers is sufficient to guide cellular arrangement.

This outcome is noteworthy as it addresses the limitation noted in the existing aligned fiber mat fabrication method. Most studies fabricated fibers with uniform diameters, resulting in decreased fiber mat porosity, thus hindering cellular penetration [45,46]. In contrast, this scaffold displayed aligned microscale fibers interspersed with randomly distributed nanofibers, exhibiting enhanced porosity compared to traditionally aligned fibers with uniform diameters. Such structures can provide aligned topological cues with porosity, benefiting various tissues, including muscle [47], vascular [48], nerves [49], and even bone [50].

## 4. Conclusions

This study provided several lines of evidence for the versatile applications of PαAPz-M in tissue engineering. As evidenced by the DPPH assay, PαAPz-M has shown a significant ROS scavenging capacity, positioning it as a promising material for scavenging ROS. Additionally, PαAPz-M fiber mats have demonstrated the potential to protect cells from exogenous ROS, which could affect cell viability during maturation and implantation. In the subsequent material-directed differentiation study, it was shown that phosphate ions play a significant role in directing iMSCs toward osteogenic differentiation, and aligned fiber topology guides cell arrangement. Notably, phosphate ions are the late-stage degradation product of PαAPz-M, and early-stage degradation products such as methionine, did not show a significant influence on cell differentiation, which means PαAPz-M holds potential for applications in tissue engineering beyond bone regeneration. This research explored the functionality of PαAPz-M in both protecting cells from oxidative damage and material-directed cell differentiation and expanded the application of PαAPz-M in tissue engineering. The outlook for poly(amino acid ester) phosphazenes, such as PαAPz-M, is highly promising in the fields of tissue engineering and drug delivery. However, further research is required to overcome challenges such as electrospinnability.

## Figures and Tables

**Table 1:** Spinning parameters to produce random and aligned fibers.

	Solvent Ratio	Rotating Speed (RPM)	Voltage (kV)	Concentration (wt%)	Flow Rate (mL/h)
Random	CF:DMSO (3:1)	300	20	10	0.4
Aligned	CF:DMSO (3:1)	1500	15	15	0.8

## Data Availability

The data that support the findings of this study are available from the corresponding author, Kibret Mequanint, upon reasonable request.
